# Germinal Center Centroblasts Transition to a Centrocyte Phenotype According to a Timed Program and Depend on the Dark Zone for Effective Selection

**DOI:** 10.1016/j.immuni.2013.08.038

**Published:** 2013-11-14

**Authors:** Oliver Bannard, Robert M. Horton, Christopher D.C. Allen, Jinping An, Takashi Nagasawa, Jason G. Cyster

**Affiliations:** 1 Howard Hughes Medical Institute, University of California, San Francisco, San Francisco, CA 94143, USA; 2 Department of Microbiology and Immunology, University of California, San Francisco, San Francisco, CA 94143, USA; 3 Sandler Asthma Basic Research Center, University of California, San Francisco, San Francisco, CA 94143, USA; 4 Department of Immunobiology and Hematology, Institute for Frontier Medical Sciences, Kyoto University, Kyoto 606-8507, Japan

## Abstract

Germinal center (GC) B cells cycle between the dark zone (DZ) and light zone (LZ) during antibody affinity maturation. Whether this movement is necessary for GC function has not been tested. Here we show that CXCR4-deficient GC B cells, which are restricted to the LZ, are gradually outcompeted by WT cells indicating an essential role for DZ access. Remarkably, the transition between DZ centroblast and LZ centrocyte phenotypes occurred independently of positioning. However, CXCR4-deficient cells carried fewer mutations and were overrepresented in the CD73^+^ memory compartment. These findings are consistent with a model where GC B cells change from DZ to LZ phenotype according to a timed cellular program but suggest that spatial separation of DZ cells facilitates more effective rounds of mutation and selection. Finally, we identify a network of DZ CXCL12-expressing reticular cells that likely support DZ functions.

## Highlights

• CXCR4-deficient B cells are gradually outcompeted of influenza-induced GCs
• GC B cells transition from the DZ to LZ state according to a cellular program
• DZ access is required for normal somatic hypermutation rates
• The DZ contains a dense network of CXCL12-expressing reticular cells

## Graphical Abstract

**Figure:**
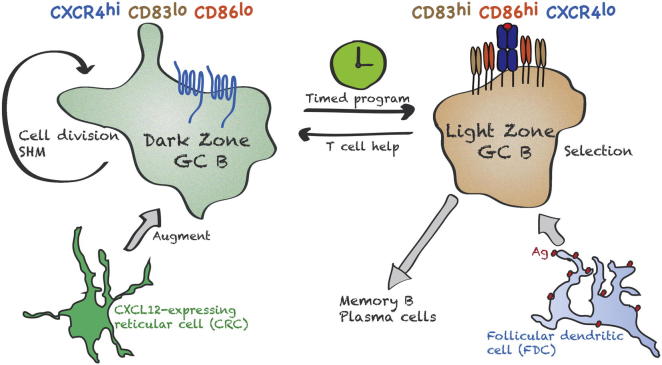

## Introduction

Germinal centers (GCs) form in secondary lymphoid organs after immunization or infection. They are the principal sites in which B cells modify their immunoglobulin (Ig) variable genes by somatic hypermutation (SHM) and undergo selection for increases in Ig affinity for antigen. It has been recognized for more than 80 years that the GCs are polarized into two zones, the dark zone (DZ) and the light zone (LZ) (Rohlich, 1930). GC B cells in the DZ and the LZ are termed centroblasts and centrocytes, respectively. Although initially described based upon histological observations of its lower B cell density, the LZ is also distinguished by the presence of follicular dendritic cells (FDC) that express high amounts of the complement receptors CD21 and CD35 and FcγRII (CD32) that capture and display immune complexes, and by its containing the majority of GC follicular helper T cells (Tfh) that provide help to B cells. Both of these LZ resident accessory populations are critical to GC responses (Victora and Nussenzweig, 2012; Wang et al., 2011). GC polarization is conserved across a range of species (Allen et al., 2004; Victora et al., 2012; Yasuda et al., 1998), strongly suggesting that it plays an important role; however, this has not been carefully tested and the function of the DZ is not clear.

Recent advances in imaging have allowed visualization of GC cell behavior in vivo and have established that GCs are highly dynamic structures in which B cells transit back and forth between zones (Allen et al., 2007b; Victora et al., 2010). The rapid exchange of cells between compartments suggests that centroblasts and centrocytes might be better considered as different transient states within the same developmental step, rather than being different stages of differentiation. This conclusion is further supported by findings that centroblasts and centrocytes are indistinguishable in terms of size and morphology and that there is great overlap in their gene-expression profiles (Allen et al., 2007b; Victora et al., 2010). Nevertheless, centroblasts and centrocytes do differ in expression of a range of genes involved in activation, chemokine responsiveness, DNA repair, and proliferation (Allen et al., 2004; Victora et al., 2012). Therefore, we continue to use the centroblast and centrocyte nomenclature but define these states based on expression levels of the “signature” surface proteins CXCR4, CD83, and CD86; centroblasts express higher amounts of CXCR4 but lower amounts of CD83 and CD86, whereas centrocytes are identified as being CXCR4^lo^, CD83^hi^, and CD86^hi^ (Allen et al., 2004; Victora et al., 2010). It is thought that these changes in phenotype are the outcome of different local inputs within the DZ and LZ, but this has not been tested (Victora et al., 2012). In contemporary models of the GC response, SHM and proliferation occur in the DZ and are followed by B cell shuttling to the LZ where antigen is captured through their newly mutated BCRs and internalized for presentation to T cells (Allen et al., 2007a; Victora and Nussenzweig, 2012). B cells with the highest affinity acquire more antigen and present more peptide-MHC class II complexes on their surface, enabling out-competition of their neighbors (Allen et al., 2007b; Victora et al., 2010). Iterative rounds of mutation and selection lead to affinity maturation at the population level.

GC organization requires expression by B cells of the chemokine receptors CXCR5 and CXCR4 (Allen et al., 2004). The ligand for CXCR5, CXCL13, is expressed by FDC in the LZ and is responsible for guiding migration to this compartment, whereas transit to the DZ and away from CXCL13 is dependent upon centroblasts expressing greater amounts of CXCR4 on their surface. CXCR4 deficiency in small fractions of GC B cells leads to their sequestration in the LZ. Here we took advantage of this requirement to explore the role of the DZ in GC responses. Surprisingly, the transition from centroblast to centrocyte phenotype does not depend on unique zonal cues. However, access to the DZ is critical for effective participation within the GC; CXCR4-deficent cells acquire fewer mutations and are outcompeted over time. We propose that this reflects a defect in selection when SHM and antigen acquisition are not spatially separated. Finally, we report that the DZ contains a dense network of stromal cells expressing CXCL12 (the ligand for CXCR4), and we suggest that these cells help support GC responses.

## Results

### CXCR4 Is Required for Effective Competition in GCs

**Figure 1:**
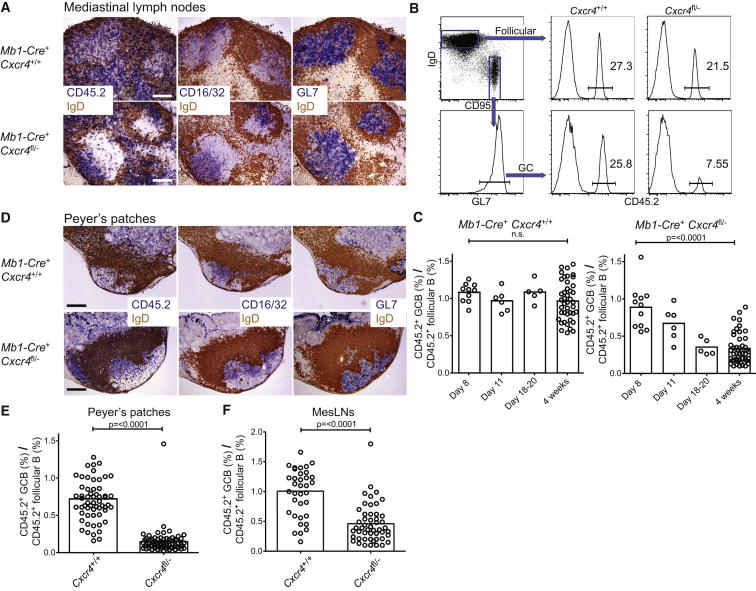
CXCR4 Is Necessary for DZ Access and Continued Participation in Influenza and Gut Associated GCs (A) Mixed BM chimeric mice with a majority of CD45.1^+^ and a minority of CD45.2^+^*Mb1-Cre*^+^*Cxcr4*^fl/−^ or CD45.2^+^*Mb1-Cre*^+^*Cxcr4*^+/+^ control B cells were infected with HKx31 influenza, and medLNs were harvested for IHC analysis on day 13 p.i. (B) Gating scheme for assessing the participation of CXCR4-deficient B cells within the follicular and GC compartments, showing example from day 28 p.i. (C) Data from multiple experiments were plotted as percentage of CD45.2^+^ GC B cells/% within the concurrent follicular compartment, at various time points. (D–F) Similar IHC (D) and FACS (E and F) analysis was performed for PPs and mesLNs. Dots in (C), (E), and (F) represent single animals, and error bars indicate means. Comparisons use a nonpaired two-tailed Student’s t test. Scale bars represent 200 um. See also Figure S1.

To test whether DZ access is critical for GC responses, we generated mixed bone-marrow (BM) chimeric mice where a majority of B cells were from wild-type (WT) CD45.1^+^ donor mice, but that also contained a smaller fraction (∼10%–40%) of B cells lacking CXCR4 (CD45.2^+^*Mb1-Cre*^+^*Cxcr4*^fl/–^). Chimeric mice were infected with the HKx31 strain of influenza virus and responses were followed in the draining mediastinal LNs (medLNs). The presence of large numbers of WT GC B cells permits the development of normal GC structures in which the CXCR4-deficient cells can compete but are restricted to the LZ (Figure 1A; see also Figures S1A and S1B available online) (Allen et al., 2004). Small numbers of CD45.2^+^ cells were found in the DZ of these GCs, but these were mostly T cells (Figure S1C). In the control mixed chimeras, CD45.2^+^ *Mb1-Cre*^+^ *Cxcr4*^+/+^ B cells were present throughout the GC, as expected (Figure 1A; Figure S1C). FDC organization and polarization, as determined by CD16/32 staining, was normal in both sets of mice (Figure 1A; Figure S1A).

The contribution of CXCR4-deficient cells to the IgD^lo^ CD95^hi^ GL7^+^ GC population within the medLN was determined at various time points after infection and compared to the concurrent naive follicular compartment (Figure 1B). Early in the response on day 8 postinfection (p.i.), the proportions of GC and follicular cells that were CXCR4-deficient was almost identical, indicating that B cells do not require responsiveness to CXCL12 for seeding the response (Figure 1C). However, the *Cxcr4*^fl/−^ population gradually shrunk as a proportion of the GC over time, with their representation in the GC at 4 weeks p.i. being approximately a third of that in the follicular compartment at the same time point (Figures 1B and 1C), indicating a requirement for CXCL12 responsiveness to compete effectively within these GCs. Importantly, no defect was seen in control mixed chimeric mice.

We performed a similar analysis of GC responses in the Peyer’s patches (PPs) and mesenteric LNs (mesLNs) of mixed BM chimeric mice, where chronic GCs form in response to gut-associated antigens. We again observed *Cxcr4*^fl/−^ GC B cells to be restricted to the LZ when we looked in PPs (Figure 1D) and saw similar (mesLN, Figure 1F) or slightly stronger (PPs, Figure 1E) defects in GC participation when cells could not access the DZ. Therefore, a requirement for CXCR4-expression by GC B cells is not specific to influenza infection or to the medLN.

### CXCR4-Mediated DZ Access Is Essential for Effective GC Responses

CXCR4 ligation in vitro can lead to Ca^2+^ flux and the activation of ERK and AKT (Busillo et al., 2010). Therefore, where CXCR4 is required for effective responses in several lineages, it has been difficult to elucidate whether the chemokine dictates cell fate through the regulation of positioning or through its direct activation of these intracellular signaling pathways. This is particularly true where CXCL12 is reported to promote proliferation and differentiation processes in vitro, because chemotactic responses should not factor in these assays, but their relation to in vivo observations is not clear.

**Figure 2:**
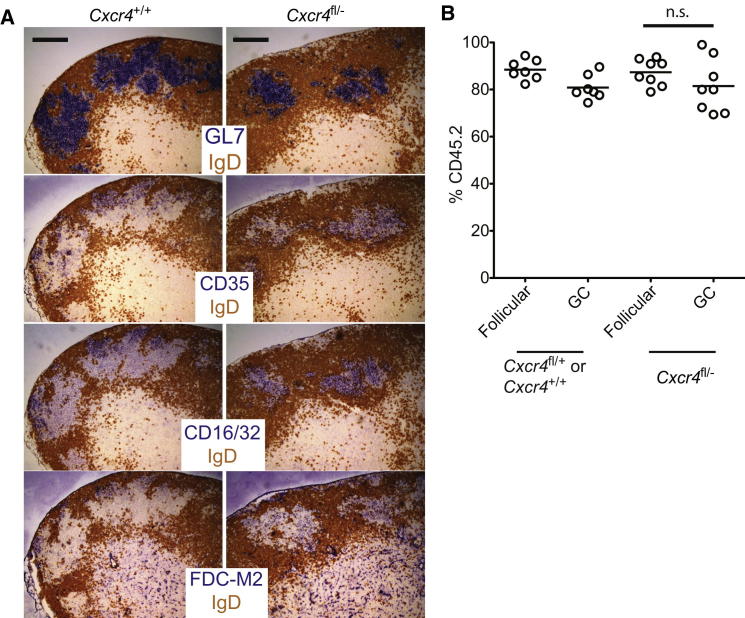
CXCR4 Expression Is Not Required for Effective Competition in Nonpolarized GCs BM chimeric mice were generated in which the majority (75%–95%) of B cells were CXCR4-deficient (or control), and infected with influenza virus. (A) GC polarization was determined by IHC staining of medLNs on day 12. (B) Participation of CD45.2^+^*Mb1-Cre*^+^*Cxcr4*^fl/−^ or control cells within follicular B cell and GC compartments at 4 weeks p.i., with each dot representing a single mouse, and error bars indicating means. Comparisons were with a paired two-tailed Student’s t test. Scale bars represent 200 um.

In an effort to gain insight into whether CXCR4-expression by GC B cells has a role beyond promoting DZ access, such as directly stimulating prosurvival or proliferation pathways, we generated mixed BM chimeric mice in which the majority (75%–95%) of follicular B cells were from CD45.2^+^ *Mb1-Cre*^+^ *Cxcr4*^fl/−^ (or *Mb1-Cre*^+^ *Cxcr4*^+/+^) mice, and a minority were from CD45.1^+^ WT donors. Because GCs lack proper stromal polarization when most GC B cells lack CXCR4 (Allen et al., 2004), this setup allowed us to drive small numbers of WT GC B cells into nonpolarized GCs (Figure 2A). We determined participation by CXCR4-deficient and sufficient cells within these abnormal GCs at 4 weeks p.i. and found the frequencies of each population to match that in the concurrent follicular population, indicating that CXCR4-expression was not required for participation in these nonpolarized GCs (Figure 2B). These findings are dramatically different from those in polarized GCs where CXCR4 expression was required for the cells’ continued participation (Figure 1) and argue that expression of CXCR4 by GC B cells promotes effective responses only when it regulates entry into the DZ.

### Cycling between Centroblast and Centrocyte Stages Does Not Require DZ Access

**Figure 3:**
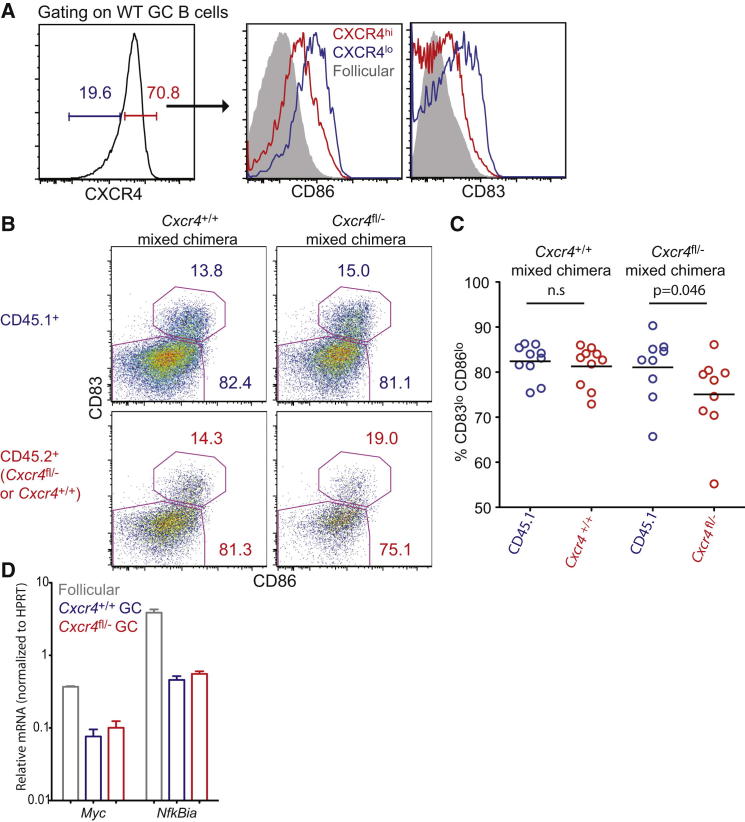
Aspects of GC B Cell Behavior Are Regulated Independently of Cues within a Particular Zone (A) IgD^lo^CD95^+^GL7^+^ WT GC B cells from the medLNs of influenza infected mice were costained for surface CXCR4 expression and for the LZ markers CD83 and CD86 at ∼4 wks p.i. (B) Representative plots of CD83 and CD86 staining (mean %s) on *Cxcr4*^fl/−^ and control GC B cells from mixed chimeric mice at 4 weeks p.i. (chimerism as in Figure 1 and Figure S1). Data were pooled from several experiments, with each dot in (C) representing a single mouse. (D) qPCR measurements of mRNA levels in FACS sorted IgD^lo^CD95^+^GL7^+^*Cxcr4*^fl/−^ and WT GC B cells from mixed BM chimeras. Data in (D) are pooled from two to three mice with error bars indicating SD. Comparisons use a paired two-tailed Student’s t test. See also Figure S2.

To determine whether zone-restricted cues cause the centrocyte and centroblast states of GC B cells, we compared CD83 and CD86 expression by CXCR4-deficient and CD45.1^+^ WT GC B cells from within the same LN when only WT cells might access the DZ (Figure 1A). Like in NP-OVA immunized mice (Victora et al., 2010) and in human tonsils (Victora et al., 2012), we saw more CD83 and CD86 on WT CXCR4^lo^ centrocytes than on CXCR4^hi^ centroblasts during influenza infection (Figure 3A). Because we could not use CXCR4 as a marker to distinguish centrocytes and centroblasts of CXCR4-deficient origin, we determined the frequencies of cells in these states based on CD83 and CD86 expression (Figure 3B). To our surprise, the frequency of CXCR4-deficient GC B cells displaying a centroblast (CD83^lo^CD86^lo^) phenotype was very similar to that of WT with just a slight shift toward more centrocyte stage cells (Figures 3B and 3C), despite their severe impairment in accessing the DZ (Figure 1A). We also saw quite normal frequencies of centroblasts and centrocytes in mice lacking CXCR4 on all B cells, where GCs are not polarized and therefore do not have a discernible DZ (Figures S2A–S2C). In keeping with these findings, transcripts for the LZ cell genes *Myc* and *Nfkbia* (Victora et al., 2012; Victora et al., 2010) were similarly abundant in WT and *Cxcr4*^fl/−^ GC B cells in mixed BM chimeric mice (Figure 3D). Together, these findings suggest that expression of centrocyte-associated genes increases without requiring access to DZ-restricted cues and presumably coinciding with the decrease in surface CXCR4, though access to the DZ has a small augmenting influence on acquisition or maintenance of a centroblast phenotype.

### Cell-Cycle Progression within the LZ

**Figure 4:**
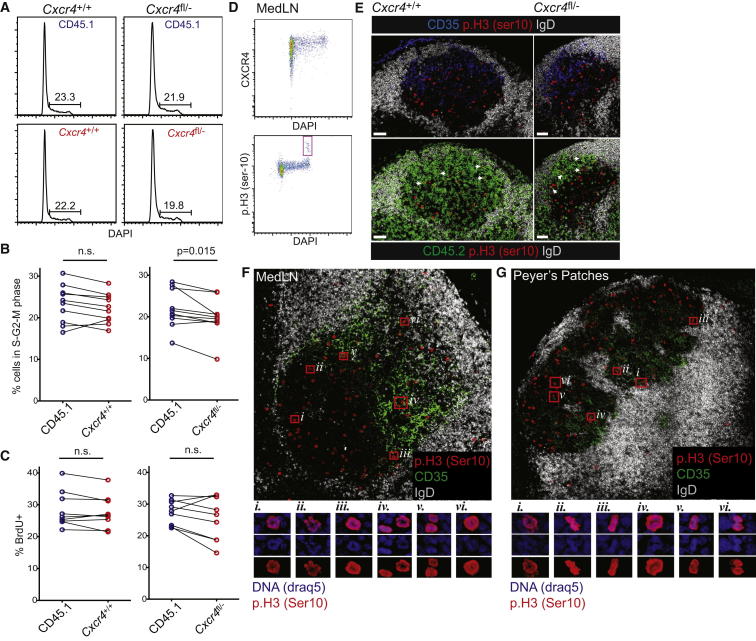
DZ Access Is Not Required for Cell-Cycle Progression or Mitosis (A and B) DNA content in *Cxcr4*^fl/−^ and control GC B cells from mixed BM chimeras (as in Figure 1) was assessed at ∼4 weeks after influenza infection (A). Data from multiple experiments are represented in (B). (C) Proliferation was determined after a single BrdU injection 30 min prior to euthanasia. Lines in (B) and (C) join CD45.1^+^ WT and CD45.2^+^*Cxcr4*^fl/−^ or control cells from the same mouse. (D) GC B cells from influenza-infected WT mice were assessed by FACS for p.H3, DNA content, and CXCR4 expression. Red box highlights p.H3 high cells. (E) MedLNs from influenza infected mixed BM chimeras were stained at 4 weeks p.i. for p.H3, CD35, IgD, and CD45.2. Arrows indicate cells in the LZ that are double positive for CD45.2 and p.H3. (F and G) p.H3 staining in WT medLN (F) and PP GCs (G). Symbols and red boxes identify the high magnification regions shown below. Comparisons are with a paired two-tailed Student’s t test. Scale bars represent 50 um.

We examined whether cues in the DZ regulate mitosis by comparing the frequency of CXCR4-deficient and WT GC B cells in S-G2-M phases of cell cycle. By DNA content measurement, similar frequencies of *Cxcr4*^fl/−^ (19.8%) and WT cells (21.9%) were actively proliferating at the time of analysis (Figure 4A), although the proportion of cells in S, G2, and M was marginally lower for the CXCR4-deficient cells (p = 0.015) (Figure 4B). This difference was small and only significant when data from all mice were pooled and compared, but not when the means from the four experiments were compared. A similar trend, but one that did not reach statistical significance, was observed when bromodeoxyuridine (BrdU) incorporation over a 30 min period was compared (Figure 4C).

We further examined the sites in which CXCR4-deficient cells were undergoing mitosis in situ. Histone H3 is rapidly phosphorylated at Ser-10 as cells progress from late G2 into prophase, with dephosphorylation occurring by the anaphase to telophase step (Dai et al., 2005). Therefore, unlike BrdU staining, positivity for phosphohistone H3 (p.H3) identifies mitotic but not S phase cells (Figure 4D). CD45.2^+^ p.H3^+^ cells were found almost exclusively within the LZs of *Cxcr4*^fl/−^ mixed chimeric mice, confirming that cellular division was occurring in that zone (Figure 4E). We also saw significant numbers of p.H3^+^ cells in the CD35^hi^ LZs of control WT mice, despite G2-M phases of cell cycle being mostly restricted to cells at the CXCR4^hi^ stage (Figure 4D) (Allen et al., 2007b; Victora et al., 2010). Similar patterns of p.H3 staining were observed in medLNs and PPs of WT mice (Figures 4F and 4G), indicating that this phenomenon is not a consequence of earlier exposure to radiation and was not unique to the antiviral response. Our confidence in the specificity of the staining reagent was further enhanced by the observation that p.H3^+^ cells frequently displayed signs of condensed chromatin and did not appear associated with tingible body macrophages. Taken together, these results indicate that GC B cell progression through cell cycle does not depend upon access to DZ cues and highlights how the spatial separation of centroblast and centrocyte functions is not absolute even in WT GCs.

### Lower Accrual of Mutations in CXCR4-Deficient GC B Cells

**Figure 5:**
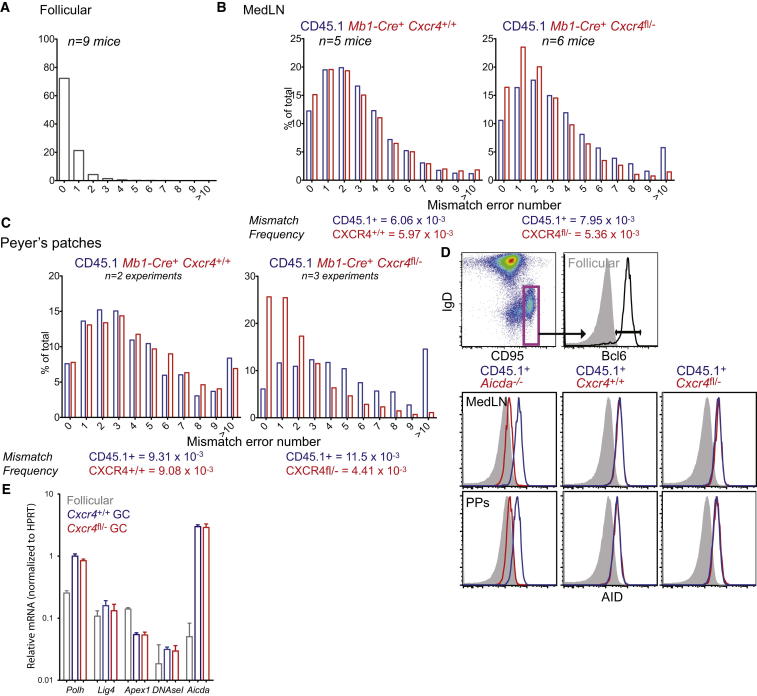
Reduced Mutation Load in CXCR4-Deficient GC B Cells Follicular (A) and GC (B) B cells from medLNs of CXCR4-deficient and control mixed BM chimeric mice were FACS isolated on days 23–25 of influenza infection. The frequency of mismatch errors in 470 bp of the intron downstream of rearranged VDJ regions was determined by single molecule sequencing. CD45.2^+^*Cxcr4*^fl/−^ and control cells were compared to WT CD45.1^+^ cells from the same mice. n = number of pooled mice. (C) Similar analysis for PPs. Approximately three mice were pooled for each PP experiment, and data are pooled from indicated number of experiments. (D) Intracellular AID protein staining of medLNs from mixed BM chimeras at day 15 p.i. *Aicda*^*−/−*^ cells and WT CD45.1^+^ cells were mixed to provide staining controls. The GC gating scheme is shown above. Representative of six mice from three experiments. (E) RT-PCR measurements of genes associated with SHM for *Cxcr4*^fl/–^ and WT cells from mixed BM chimeras. Data are pooled from two to three mice with error bars indicating SD. See also Figure S3.

To determine whether SHM within the responding polyclonal population of *Cxcr4*^fl/−^ GC B cells was impacted by their restriction to the LZ, we isolated B cells from mixed chimeras and sequenced a 470 bp region from the JH4 intronic region of rearranged members of the abundant VH558 V-region family by using Pacific Biosciences single-molecule DNA sequencing technology. The mutation frequency in this intronic region provides a measurement of AID activity (Jolly et al., 1997). An analysis of the mismatch error rate in follicular B cells as a negative control for AID activity confirmed the appropriateness of the sequencing platform; the error rate was comparable to that expected from nested PCR alone (Figure 5A).

CXCR4-deficient or control GC B cells were compared to WT CD45.1^+^ cells from the same medLNs 23–25 days after influenza infection. While the frequency of mutations in *Mb1*-*Cre*^+^ *Cxcr4*^+/+^ and CD45.1^+^ WT cells was similar in control mice, *Cxcr4*^fl/−^ GC B cell populations from four of six mice contained fewer mutations. This trend is shown in Figure 5B, which displays the mean data set. Analysis of PP GC B cells showed a similar pattern, but with even fewer mutations in CXCR4-deficient GC B cells relative to controls (Figure 5C). Therefore, effective somatic hypermutation requires GC B cell responsiveness to CXCL12.

Activation-induced cytidine deaminase (AID) is thought to be more abundant in DZ than in LZ cells (Victora et al., 2012). We optimized a FACS-based assay to determine AID protein levels in *Cxcr4*^fl/−^ GC B cells, to ask whether its expression might be positively regulated by cues in the DZ microenvironment. AID was equally abundant in *Cxcr4*^fl/−^ and WT GC B cells (Figure 5D). Comparisons of *Aicda*^−/−^ and WT GC B cells confirmed the specificity of the antibody stain (Figure 5D). Furthermore, *Polh*, *DnaseI*, *Lig4*, and *Apex1* transcripts, genes involved in SHM that are also most abundant in DZ cells (Victora et al., 2012), had similar expression patterns in *Cxcr4*^fl/−^ and control GC B cells (Figure 5E). The extent and type of class-switch recombination in both populations, another AID-dependent process that can occur during pre-GC differentiation or in the GC, also did not depend upon DZ access (Figure S3). Therefore, although accumulation of normal numbers of nucleotide substitutions requires responsiveness to CXCL12, DZ access is not critical for the expression of key enzymes involved in SHM.

### Memory and Plasma Cell Development without Access to the GC DZ

**Figure 6:**
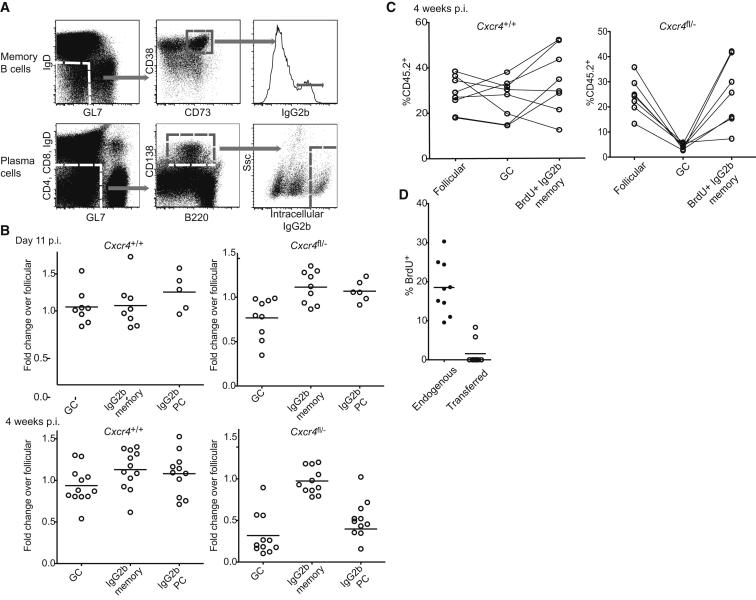
Normal PC Development but Increased Memory Cell Output when GC B Cells Are Restricted to the LZ (A) Representative FACS plots showing memory cell and PC gating schemes. (B) The frequency of CD45.2^+^*Cxcr4*^fl/−^ and control cells in mixed BM chimeric mice (as in Figure 1) was determined for the GC, memory, and PC compartments and plotted as fold difference relative to the concurrent follicular population. Mice were analyzed at 11 days and ∼4 wks p.i. Dots represent individual animals, and error bars show means. (C) Influenza-infected *Cxcr4*^fl/−^ and control mixed BM chimeric mice were given BrdU for 3 days at 4 weeks p.i. The proportions of follicular, GC, and BrdU^+^ memory cells that were CD45.2^+^ are connected for each mouse. (D) Memory cell proliferation was assessed by transferring splenocytes from 3–4 weeks influenza-infected mice into infection-matched recipients and giving recipients BrdU from 24 hr after transfer until analysis 3 days later. BrdU incorporation by transferred and endogenous IgD^lo^ GL7^lo^ CD73^+^ CD38^+^ memory cells was compared. Each dot represents a single animal, and error bars show means.

GC B cells can undergo one of two terminal fates; they might differentiate into memory cells or plasma cells (PCs). We examined whether access to the DZ was necessary for the effective transition to these stages in *Cxcr4*^fl/−^:WT mixed BM chimeric mice following influenza infection. PCs were identified by being negative or low for CD4, CD8, GL7 and IgD, as expressing intermediate levels of B220, being positive for CD138, and by their high intracellular IgG2b levels (Figure 6A). Memory cells are long-lived and might be generated in GC-dependent and independent processes; however, expression of CD73 was recently shown to identify memory cells generated via a mostly, but not exclusively, Bcl6-dependent pathway (Kaji et al., 2012; Taylor et al., 2012). We therefore used CD73 to enrich for GC-dependent memory cells within our CD38^hi^ GL7^lo^ IgD^lo^ IgG2b^+^ gating scheme. Assessments were made both early in the response on day 11 p.i, when *Cxcr4*^fl/−^ B cells show minimal defects in GC participation (Figure 1B), and 4 weeks p.i., when the competitive fitness of *Cxcr4*^fl/−^ cells has been strongly impacted. While many B220^int^ CD138^+^ intracellular-Ig^hi^ cells present at day 11 will presumably have arisen through an extrafollicular GC-independent pathway, PC phenotype cells present at the later time point should mostly be derived from GCs. At both time points, *Cxcr4*^fl/−^ PCs were present at approximately the same frequency as *Cxcr4*^fl/−^ cells in the GC compartment, suggesting that the GC-to-PC transition was not dependent upon access to the DZ.

In contrast to PCs, the frequency of CD73^+^ memory cells within the *Cxcr4*^fl/−^ population at 4 weeks p.i. was greater than expected given their participation within the GC, suggesting that they might have an increased propensity to transition from GC to memory stages (Figure 6B). To further examine this possibility, we treated mice with BrdU for 3 days prior to analysis. Memory cells might form early in responses and persist for months; however, BrdU staining should mark cells that divided within the GC during the treatment period but prior to differentiation (Anderson et al., 2007; Kaji et al., 2012). The marking of recently generated memory cells by BrdU pulsing did not affect the outcome of these experiments; CXCR4-deficient memory B cells were present at a higher frequency than GC B cells in both assays, arguing that aberrant positioning might lead to an increased output of this cell type (Figure 6C). To control for the possibility that memory B cells arriving in medLNs from another site such as the spleen were still undergoing proliferation, we performed adoptive transfer of splenocytes from 3–4 week infected donor mice in to 3–4 week infected recipient mice, placed the recipient mice on BrdU-containing water for 3 days, and then analyzed BrdU incorporation by endogenous and transferred memory B cells in medLN. This analysis established that recirculating memory cells do not proliferate within 3 days of entering the medLN (Figure 6D) and supported the suggestion that CXCR4-deficient GC B cells give rise to elevated frequencies of memory cells.

### Identification of a CXCL12-Expressing Reticular Cell (CRC) Network in the DZ

**Figure 7:**
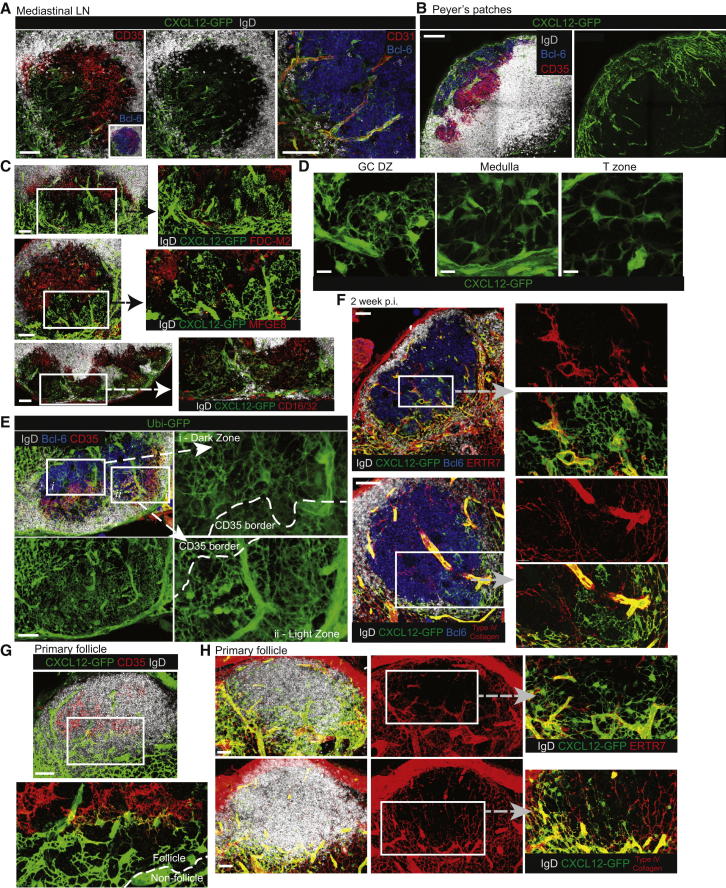
The GC DZ Contains a Network of CXCL12-Expressing Reticular Cells (A and B) Confocal microscopy of MedLNs (A) and PPs (B) from *Cxcl12-gfp* mice at day 11–12 after influenza infection. Note that the right micrograph in (A) is of higher magnification. (C) MedLNs and PPs from *Cxcl12-gfp* mice were stained for FDC markers at day 11 p.i. (D) Ubi-GFP mice were reconstituted with WT nonflorescent BM and infected with influenza. MedLNs were harvested at day 10 p.i. (E) High-powered micrographs of CXCL12-GFP^+^ stroma in the medLN GC DZ, medullary region, and T zone on day 12 p.i. (F) MedLNs from *Cxcl12-gfp* mice were stained with the indicated antibodies at 2 weeks p.i. (G and H) Peripheral skin-draining LN primary follicles from uninfected mice were stained with the indicated antibodies. Arrows indicate higher magnification images of boxed areas. Scale bar represents 10 μM (E), 30 μM (G and H) 60 μM (A, C, D, and F) and 100 μM (B). See also Figures S4 and S5 and Movie S1.

Having determined that access to the DZ is important for effective GC responses, we examined whether the DZ contains stromal cells that might provide support to GC responses. CXCL12 mRNA and protein were previously detected within the DZ (Allen et al., 2004); however, past studies examining endogenous CXCL12 were not able to define the precise location or properties of the CXCL12-expressing cells. In an effort to improve sensitivity, we used confocal microscopy to examine GFP expression in heterozygous *Cxcl12-gfp* gene targeted mice (Ara et al., 2003). This approach revealed the presence of an interconnected network of CXCL12-expressing reticular cells (CRCs) in influenza-induced GCs at various time points p.i. (Figure 7A; Figure S4A; Movie S1) and within chronic gut antigen associated PP GCs (Figure 7B; Movie S1). Within the GC, detectable GFP expression was almost entirely confined to the DZ stroma, although some DZ CXCL12-GFP^+^ cells did appear to stain weakly for CD35 (Figure 7A; Figure S4B; Movie S1). Other LZ FDC markers (CD16/32, MFGE8, FDC-M2) were undetectable or present at very low levels on DZ CRCs (Figure 7C). LZ and DZ stroma were similar morphologically; high magnification projections of DZ CRCs *in Cxcl12-gfp* mice (Figure 7D; Figure S4C) and of CD35^+^ LZ FDCs in Ubi-GFP mice that had been reconstituted with non-florescent BM (Figure 7E) revealed that both populations formed highly branched and tight reticular networks. In general, the GC stroma appeared sparse in the number of cell bodies, but it formed a dense mesh of fine processes. By contrast, CXCL12-expressing stromal cells in the T zone and medullary areas extended fewer but broader processes and often, particularly in the case of T zone FRCs, formed more regular organized structures (Figure 7D; Figure S4C). In addition to the DZ CRCs, we frequently observed CD31^+^ blood vessels ensheathed by CXCL12^+^ pericytes immediately adjacent to and within GCs (Figure 7A).

We costained DZ CRCs with antibodies that recognize antigens associated with other LN stromal subsets. In contrast to the neighboring T zone stroma, and to the follicular conduit and blood vessel-associated FRCs, DZ CRCs mostly did not express detectable levels of ERTR7 and were not tightly associated with the type IV collagen extracellular matrix (Figure 7F). These findings, together with the striking differences in morphology displayed by DZ CRCs and T zone FRCs, indicates that DZ CRCs most likely do not arise from T zone stroma that were “engulfed” when the GC expanded. We therefore asked whether CRCs might be present within naive primary follicles because such cells might contribute to early stages of GC polarization before later forming the DZ CRC network. Consistent with this possibility, we frequently observed CXCL12-GFP^+^ reticular cells in the primary follicle close to where it meets the T cell or medullary zone (Figure 7G). This primary follicle CRC network often extended to the edge of the CD35^+^ FDC network deeper within the follicle, where it sometimes appeared that there was overlap in GFP and CD35 distribution, perhaps reflecting both stromal cell types receiving similar inductive signals at the transitional area (Figure 7G). Like DZ CRCs, primary follicle CRCs extended fine processes from their globular body and they were mostly negative for ERTR7 (Figure 7H), although they did show some association with type IV collagen (albeit less consistently than for T zone FRC) (Figure 7H; Figure S5) and differed from DZ CRCs in usually not appearing to form the same tight nest-like structures.

In summary, these observations establish the presence of CXCL12-expressing reticular cell (CRC) networks within primary follicles and the GC DZ. The DZ CRCs are morphologically similar to FDCs but have a distinct chemokine expression profile and lack most FDC-associated surface markers.

## Discussion

This study provides evidence of an essential role for the DZ in the GC reaction. A major difference between the LZ and DZ is the presence in the LZ of immune-complex-decorated FDC and the majority of GC Tfh cells. The cues that promote the transition of centroblasts to centrocytes had not been examined in detail before now, but the increased expression by centrocytes of genes commonly associated with acute activation was thought to suggest exposure of centroblasts to centrocyte-inducing signals upon arrival in the LZ (Victora and Nussenzweig, 2012). However, our data are not consistent with such a model because we found nearly normal proportions of CXCR4-deficient GC B cells displaying a CD83^lo^ CD86^lo^ centroblast-like phenotype, despite their sequestration in the LZ. Furthermore, access to the DZ was not essential for efficient passage through the S to G2-M checkpoint of the cell cycle. Together, these findings strongly suggest that centroblast and centrocyte functions are limited to a certain cellular stage, rather than to a particular site. We propose a model where switching from the centroblast proliferative stage to the centrocyte selection stage progresses according to a cellular “timer” that operates independently of DZ-derived signals. Activation of the timer might be triggered by LZ-derived signals such as receipt of T cell help. According to this model, decreases in surface CXCR4 expression by GC B cells are coordinated with reductions in proproliferative and SHM gene expression and increases in CD83 and CD86. This phenotype transition causes the cell to migrate into the LZ, rather than occurring as a consequence of it. Expression of centrocyte-associated genes (such as CD86) might change the nature of the interaction with T cells, thereby limiting the capacity for positive selection to the period after the current round of mutation and clonal expansion is complete.

While the decision of when to exit the DZ centroblast stage might be set by a cellular timer, regulation of reentry into the centroblast compartment probably involves cells testing their new BCRs by competing for the formation of productive T cell interactions (Allen et al., 2007b). Delivery of peptide antigen to a subset of GC B cells was sufficient to stimulate increased cellular division, centroblast differentiation, and ongoing GC participation at the expense of their neighbors (Victora et al., 2010). Two recent studies indicate that c-Myc might play an important role in DZ cyclic reentry because it is required for continued participation in the GC, and it is expressed by a small fraction of centrocytes that are enriched for high affinity Ig rearrangements and that have recently entered the synthesis phase of cell cycle (Calado et al., 2012; Dominguez-Sola et al., 2012). By supporting a model in which T cell-derived signals induce transient c-Myc expression, leading to reentry to the DZ stage, these studies seem consistent with transient c-Myc expression being involved in resetting the centroblast “timer.” While we emphasize the role of a zone-independent and thus likely intrinsic cellular program in coordinating the centroblast gene expression profile and in regulating the transition back to the centrocyte stage, extrinsic factors such as the nature of earlier T cell interaction might imprint aspects of behavior while in the DZ-associated state.

Although DZ access is not essential for centroblast differentiation and proliferation, it is required for effective competition and continued participation within the GC. We propose two non-mutually exclusive models that might explain this. First, the polarized GC might reflect a need for the temporal and spatial separation of centrocyte and centroblast functions, rather than cues in each zone promoting them. The most compelling example for this might be the physical separation of SHM and selection; this might facilitate complete exchange of existing BCR with newly encoded protein from the mutated locus prior to antigen and T cell exposure. Premature entry into the LZ might drive aberrant BCR signaling or lead to inappropriate T cell interactions that could result in negative selection (clonal deletion) or premature positive selection. For example, failure to upregulate CD86 following BCR crosslinking in anergic B cells leads to their killing by FasL-expressing T cells (Rathmell et al., 1998), and the low CD86 expression by centroblasts might cause these Fas^hi^ cells to suffer a similar fate following antigen presentation in the GC LZ. Over time, improper GC B cell selection would manifest as a decrease in somatic mutation accruement. As a second possibility, the activity of the SHM machinery might be bolstered by cues only present in the DZ. We did not observe differences in AID mRNA or protein abundance in CXCR4-deficient cells; however, these measures do not exclude the possibility that SHM activity is lower; its regulation is particularly stringent and includes transcriptional, posttranscriptional, and posttranslational mechanisms (McBride et al., 2004). Less efficient acquisition of nucleotide substitutions would be expected to lead to less frequent improvements in affinity and reduced competitiveness in the GC.

A key function of the GC is to generate memory B cells and PCs that provide protection against future infections of the same or similar kinds. We found that the frequency of CXCR4-deficient PCs closely matched that of the concurrent GC population, suggesting that differentiation toward this fate was not negatively affected by their inability to respond to CXCL12. This finding was true regardless of whether we looked early in the response, when many antibody-secreting cells will be generated via an extrafollicular response, and after 4 weeks of infection when most PCs should be generated in the GC. However, it must be noted that we could not assess the efficiency of generating long versus short-lived PCs because CXCR4 is required intrinsically within PCs for BM homing (Hargreaves et al., 2001; Nie et al., 2004). In contrast to PC generation, the representation of CXCR4-deficient cells within the memory compartment was greater than expected given their GC participation defect, consistent with the idea that a transition to the memory cell fate might be favored when cells cannot access the DZ. It is important to note that currently available tools do not allow for the definitive identification of memory cells that have recently exited the GC reaction, especially during complex polyclonal responses; however, work has suggested that costaining with CD73 and BrdU should enrich for such a developmental stage (Anderson et al., 2007; Kaji et al., 2012; Taylor et al., 2012). The signals that cause more cells to adopt this fate are not clear, but we think it might again be a consequence of CXCR4-deficient GC B cells engaging with T cells prematurely or aberrantly due to their positioning in the antigen and T cell rich LZ. Previous studies have indicated that the selection checkpoint for memory cell generation might be less stringent than it is for PCs (Smith et al., 2000; Victora et al., 2010). An increase in memory cell generation has been reported in at least two other settings where the B-T interaction is abnormal; in mice lacking interleukin-21R (IL-21R) specifically on B cells (Zotos et al., 2010) and in mice lacking Fas on all class-switched B cells (Hao et al., 2008). Increased memory cell numbers were also seen in *Bcl6* heterozygous mice, which might be analogous to where T cell-derived IL-21 cannot drive the maintenance of Bcl6 protein levels (Kaji et al., 2012) (Linterman et al., 2010). Therefore, we speculate that a possible increase in memory cell generation by CXCR4-deficient B cells might reflect their receiving a quality of T cell help that is sufficient to rescue them from deletion but that cannot drive their PC differentiation or stimulate their continued participation in the GC.

GC B cells are acutely dependent upon trophic factors present in their microenvironment; cells displaced from that setting die within a matter of hours (Wang et al., 2011). While LZ FDCs provide one source of such factors, our confocal analysis of CXCL12-GFP and of Ubi-GFP mice revealed the DZ to contain a patchwork of tight and highly branched reticular cells that might also contribute. Previous studies had noted some VCAM-1, fibrinogen, and CD35 staining in this zone but had not examined the nature of the stromal network in detail (Allen and Cyster, 2008). We observed some variation in DZ coverage by CXCL12-producing stroma; in some GCs, the network extended throughout the CD35^lo^ region but formed patches of “labyrinth-like” structures, seemingly similar morphologically to the reticular network described in human basal LZs (Imal and Yamakawa, 1996). LN GCs mostly form with the LZ proximal to the subcapsular sinus from where antigen drains and the DZ expands to border the neighboring T or medullary compartment. This raised the possibility that CXCL12-expressing reticular cells (CRCs) in the DZ arise by the GC engulfing the neighboring compartment as the follicle expands. However, we also identified a T-zone proximal network of CXCL12-expressing reticular cells in primary follicles. Like DZ CRCs, primary follicle CRCs were mostly distinct from neighboring FRCs both in their morphology and in their not strongly associating with ERTR7 staining, although it should be noted that both primary and secondary follicles also contained ERTR7^+^ CXCL12-GFP^+^ perivascular cells. We therefore think it more likely that preexisting follicular CRCs help to establish early stages of GC polarization and later form DZ CRCs as the GC matures. An important question for future study is the relative contributions of CRCs, CXCL12-expressing perivascular cells, and GC-proximal FRCs to GC organization and maintenance. In this regard, it is interesting to note that CXCL12-expressing perivascular and endothelial cells are important in organizing and maintaining hematopoietic stem cell and committed B cell progenitor niches in the BM (Tokoyoda et al., 2004). In this setting, cells compete for cues derived from the stromal network. It will be interesting to see whether a similar fitness competition plays out in the GC.

In summary, we have provided evidence that centrocyte differentiation from centroblasts occurs as part of a timed cellular program. We believe a key function of this program is to temporally separate the processes of SHM and mitosis from selection; as the molecular program transitions the cell to the centrocyte stage, proliferation and mutation genes are reduced and the cell readies itself for optimal engagement with antigen and with T cells. Therefore, much of GC behavior that has traditionally been described as reflecting a function of the LZ or the DZ instead occurs as part of the particular stage of the GC B cell program. However, we also provide strong evidence that the spatial separation of LZ and DZ functions is critical for maintenance of effective GC responses.

## Experimental Procedures

### Mice and Infections

Mixed BM chimeric mice were generated by transferring ∼3 × 10^6^ cells from the following mixes into lethally irradiated (2 × 450 rads, 3 hr apart) B6-CD45.1^+^ recipients: *Mb1-Cre*^+^ *Cxcr4*^fl/−^:CD45.2^+^ at 45:55, *Mb1-Cre*^+^ *Cxcr4*^+/+^:CD45.1^+^ at 25:75, *Mb1-Cre*^+^ *Cxcr4*^fl/−^:CD45.1^+^ at 5:95 and *Mb1-Cre*^+^ *Cxcr4*^+/+^:CD45.1^+^ at 85:15, to give ratios of ∼20:80, 27:75, 10:90, and 10:90, respectively. Mice lacking CXCR4 on all B cells were generated with *Mb1-Cre*^+^ *Cxcr4*^fl/−^ BM, and mice expressing GFP in all stroma were made with WT BM and Ubi-GFP recipients. Reconstituted mice were rested >8 weeks prior to infection. *Mb1-Cre*, *Cxcr4*^fl/fl^, and *Cxcr4*^−/−^, Ubi-GFP, and *Aicda*^−/−^ mice were all backcrossed to the C57BL/6 background for >8 generations (Hobeika et al., 2006; Muramatsu et al., 2000; Nie et al., 2004; Schaefer et al., 2001; Zou et al., 1998*). Cxcl12-gfp* gene-targeted mice were on an impure C56BL/6 background (Ara et al., 2003).

For influenza infections, anesthetized mice were given 2 × 10^4^ pfu of the A/HK-x31 (x31, H3N2) virus via the intranasal route. For memory B cell transfers, splenocytes (entire spleen) from infected C57BL/6 mice were transferred into B6-CD45.1^*+*^ recipients by i.v. injection. For BrdU experiments, mice received a single i.p. injection of 2.5 mg BrdU (Sigma Aldrich) 30 min prior to euthanasia or were fed water containing 0.8 mg/ml BrdU for longer-term labeling. Animals were housed in a specific pathogen-free environment at UCSF, and all experiments conformed to ethical principles and guidelines approved by the Institutional Animal Care and Use Committee of UCSF.

### Flow Cytometry

For most experiments, single cell suspensions were generated and stained as previously described using Abs listed in Table S1 (Allen et al., 2007b). For PCs, tissues were finely chopped then digested shaking for 35 min at 37°C in 0.5 mg/ml type 4 collagenase (Worthington Biochemical Corp.) in DMEM, 2% FBS, 1% HEPES. EDTA (10 mM final) was added a further 5 min. Stromal cell suspensions were prepared as described (Yi et al., 2012). For cell-cycle analysis, cells were stained with antibodies prior to fixation overnight on ice in 1% PFA in PBS. Fixed cells were washed twice with BD CytoPerm. DAPI was added to a final concentration of 5 nM in perm buffer prior to FACs on Lo setting. Doublets were excluded with Fsc and Ssc properties and by CD4 and CD8 dump-gating. BrdU staining was performed as per manufacturer’s guidelines (BD PharMingen). For AID staining, cells were fixed (30 min) and permeabilized (overnight) with eBioscience FoxP3 staining buffer. Anti-AID was preadsorbed in 2% mouse serum prior to staining in perm buffer for 1 hr at RT. Secondary stain of bio donk anti-rat was followed by 30 min incubation with 4% rat and mouse serums. Subsequent surface and Bcl6 staining was performed in perm buffer for 1 hr at RT in the presence of serums. To detect p.H3 by FACs, we fixed cells on ice in 1.6% PFA for 12 min and washed them once with PBS, 2% FBS. Cells were permeabilized by adding 500 μl 70% ice-cold Etoh dropwise while vortexing. Samples were moved to −20°C and stored overnight. The following day, cells were rehydrated in FACs buffer for 10 min and washed 2× prior to staining with rabbit anti-p.H3 Ab at RT for 1 hr. Secondary (bio donk anti-rab) and tertiary stains were performed similarly. Samples were acquired and analyzed with a BD LSR II and Flowjo (Treestar).

### IHC and Confocal Microscopy

For IHC, tissues were prepared and stained as described (Allen et al., 2007b) (Table S1). Positioning within the PPs of *Cxcr4*^fl/–^ cells was determined in mice with a higher frequency of CD45.2^+^ cells than was used for FACS analysis to enable detection of otherwise rare cells. For confocal microscopy (GFP, p.H3 in WT hosts), tissues were fixed in 4% PFA in PBS for 2 hr at 4°C, washed 4–6× in PBS, then moved to 15% (30 min) and 30% (overnight) sucrose in PBS. Tissues were flash frozen in TAK tissue-mounting media the following day, and 30 uM sections were dried for 1 hr prior to staining. We blocked 30 μM sections overnight and stained them for 12–24 hr at each step in PBS with 3%–5% mouse serum, 0.1% BSA, 0.3% Triton X-100, and 0.1% NaN3. For IF detection of p.H3 and T cells, 8 μM sections were stained in PBS and mouse serum for 1–3 hr. Slides were mounted with Fluoromount-G (Southern Biotech), and images were taken with a Leica SP5 inverted microscope with 40× and 63× oil immersion objectives. Images were analyzed and processed with the Imaris software and Adobe Photoshop. Videos were compiled with Apple iMovie. IHC images were captured with Zeiss AxioObserver microscope.

### RT-PCR and Pacific Biosciences Sequencing

qPCR was performed as previously described using RNA isolated from 10,000 – 40,000 FACS sorted cells (Allen et al., 2004) (Table S2). For JH558 intron sequencing, 11,000–30,000 GC B cells were FACs sorted and DNA extracted with QIAGEN DNEasy Kit. DNA was eluted in 100 μl H_2_O and concentrated to ∼15 μl with a centrifugal evaporator for use as template for nested PCR (Table S3). New primers and reagent were added directly to 1° PCR product for the 2° reaction. Secondary reaction primers incorporated 12 unique barcodes, enabling multiplexing of six reactions on a single SMRT cell. Primers are specific for JH558 family members, and only cells with JH4 giving bands of the correct size (∼700 bp). PCR products were cut from 1.2% agarose gels and purified (QIAquick columns, QIAGEN). Yields were ∼0.5–1.5 μg DNA. Library prep/sequencing (Pacific Biosciences RS sequencer) were performed at the UCSD BIOGEM facility with circular concensus mode, 2 × 55 min movies, with standard, and later “stage start,” procedures. Sequences were reported as FASTQ files, which were analyzed with the Bioconductor package “ShortRead.” Reads were filtered by requiring that 15 bp at each end of the target region match the reference sequence perfectly and that the spacing between these matches be within 2 bp of the expected length.

### Statistical Tests

Prism software (GraphPad) was used for statistical analysis.

## Supplemental Information

Document S1. Figures S1–S5, Tables S1–S3, and Supplemental Experimental Procedures

Movie S1. DZ CXCL12-Expressing Reticular Cells PP and influenza induced GCs *in Cxcl12-gfp* mice were stained with fluorescent antibodies and examined by confocal microscopy. Movie shows a selection of representative examples. The antibodies used are indicated for each example. Related to Figure 7.
