# Germinal Center Centroblasts Transition to a Centrocyte Phenotype According to a Timed Program and Depend on the Dark Zone for Effective Selection

**DOI:** 10.1016/j.immuni.2013.11.006

**Published:** 2013-12-12

**Authors:** Oliver Bannard, Robert M. Horton, Christopher D.C. Allen, Jinping An, Takashi Nagasawa, Jason G. Cyster

## Main Text

(Immunity *39*, 912–924; November 14, 2013)

In the version of this paper originally published online, Tables S1–S3 were omitted. The Supplemental Information has now been corrected, and the journal regrets the error.

