# Supplementary material for: Germinal Center Centroblasts Transition to a Centrocyte Phenotype According to a Timed Program and Depend on the Dark Zone for Effective Selection
Source: Immunity. 2013 Nov 14;39(5):912–24. doi: 10.1016/j.immuni.2013.08.038 (PMC3828484; doi:10.1016/j.immuni.2013.08.038)
Supplement: Document S1. Figures S1–S5, Tables S1–S3, and Supplemental Experimental Procedures [file mmc1.pdf]

Immunity, Volume *39*

Supplemental Information

Germinal Center Centroblasts Transition to a Centrocyte Phenotype According to a Timed Program and Depend on the Dark Zone for Effective Selection

Oliver Bannard, Robert M. Horton, Christopher D.C. Allen, Jinping An, Takashi Nagasawa, and Jason G. Cyster

Supplemental Inventory

Supplemental Figures and Tables

Figure S1, Related to Figure 1

Figure S2, Related to Figure 3

Figure S3, Related to Figure 5

Figure S4, Related to Figure 7

Figure S5, Related to Figure 7

Table S1, Related to Experimental Procedures

Table S2, Related to Experimental Procedures

Table S3, Related to Experimental Procedures

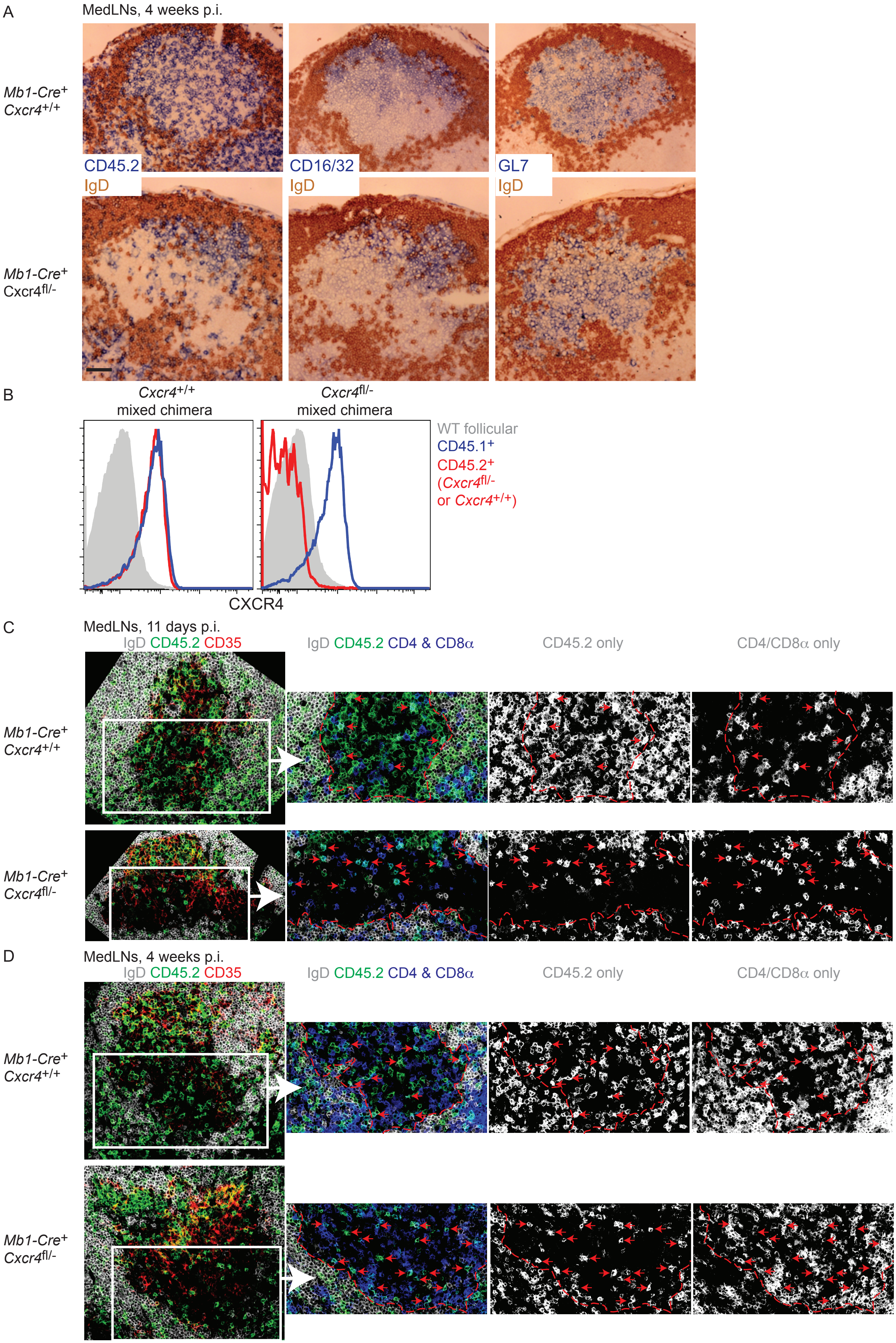

A
MedLNs, 4 weeks p.i.
Mb1-Cre+ Cxcr4+/+
Mb1-Cre+ Cxcr4fl/-
CD45.2
IgD
CD16/32
IgD
GL7
IgD
B
Cxcr4+/+ mixed chimera
Cxcr4fl/- mixed chimera
WT follicular
CD45.1+
CD45.2+
(Cxcr4fl/-
or Cxcr4+/+)
CXCR4
C
MedLNs, 11 days p.i.
IgD CD45.2 CD35
IgD CD45.2 CD4 & CD8α
CD45.2 only
CD4/CD8α only
Mb1-Cre+ Cxcr4+/+
Mb1-Cre+ Cxcr4fl/-
D
MedLNs, 4 weeks p.i.
IgD CD45.2 CD35
IgD CD45.2 CD4 & CD8α
CD45.2 only
CD4/CD8α only
Mb1-Cre+ Cxcr4+/+
Mb1-Cre+ Cxcr4fl/-

Figure S1. Efficient deletion *of Cxcr4* in GC B cells prevents their accessing the DZ, related to Figure 1.

Mixed BM chimeric mice containing a majority of WT $CD45.1^+$ and a minority of $CD45.2^+$ *Mb1-Cre*$^+$ *Cxcr4*$^{fl/-}$ or $CD45.2^+$ *Mb1-Cre*$^+$ *Cxcr4*$^{+/+}$ control B cells (as in Figure 1) were infected with HKx31 influenza. (A) The position of $CD45.2^+$ cells within a representative medLN GC was determined by IHC 4 weeks after infection. (B) CXCR4 expression by IgD^{lo} $CD95^+$ $GL7^{hi}$ GC B cells from the medLNs of mixed BM chimeras was assessed by FACS 4 weeks after infection. Data are representative of at least 6 mice. (C) Frozen medLN tissue sections from mixed BM chimeras were co-stained for T cells and assessed by confocal microscopy on day 11 p.i. and 4 weeks p.i. DZ regions identified by the white boxes in the center panels are shown at higher magnification on the right (double staining and single channel grey scale images). Dashed line shows approximate outline of follicular mantle and arrows indicate cells in the DZ that are CD45.2 and CD4 or CD8α double positive.

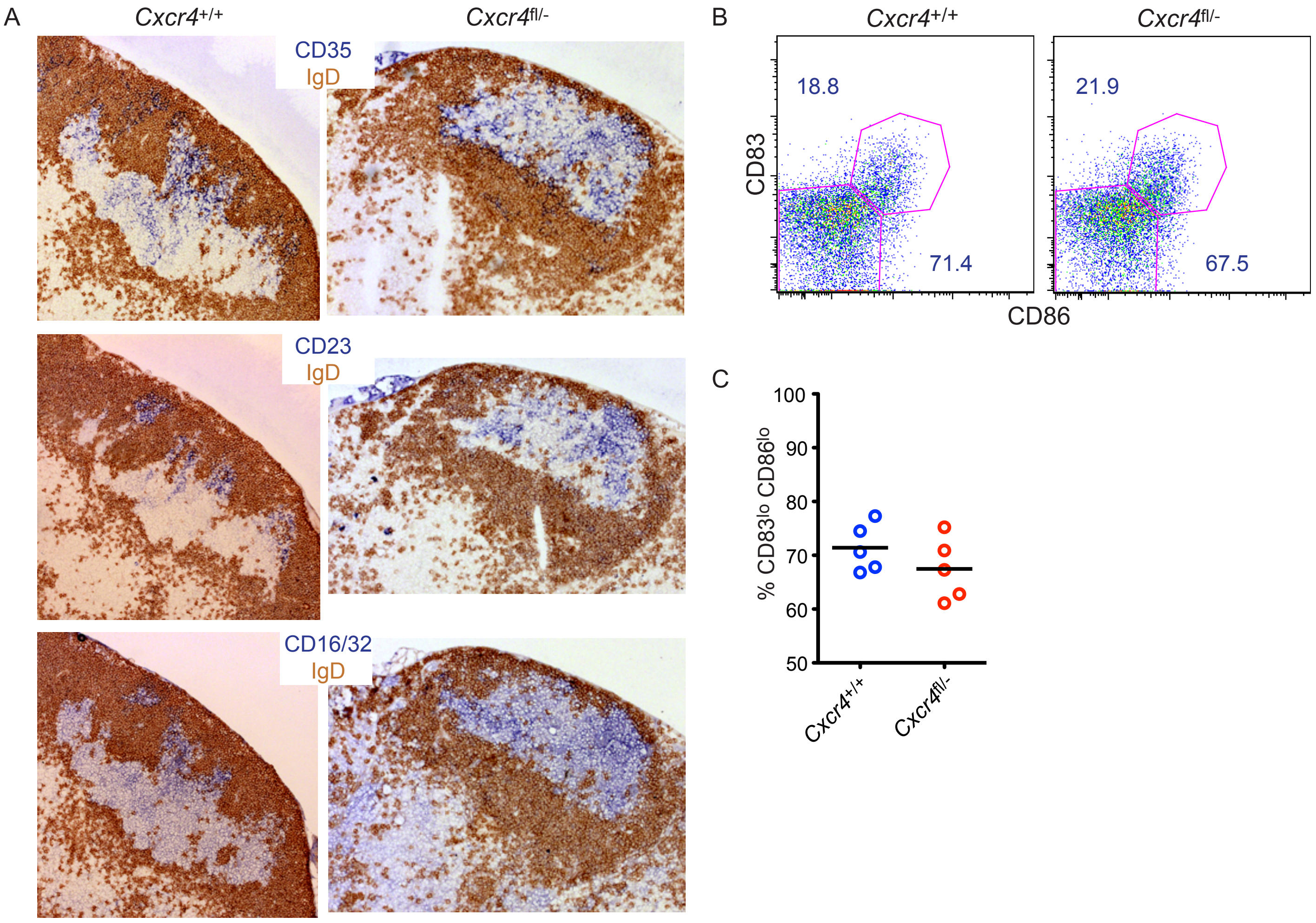
A
Cxcr4+/+
Cxcr4fl/-
CD35
IgD
CD23
IgD
CD16/32
IgD
B
Cxcr4+/+
Cxcr4fl/-
18.8
71.4
21.9
67.5
CD83
CD86
C
100
90
80
70
60
50
% CD83lo CD86lo
Cxcr4+/+
Cxcr4fl/-

Figure S2 Centroblast and centrocyte phenotype cells are both present in GCs lacking proper polarization, related to Figure 3.

Mb1-Cre^{+} *Cxcr4*$^{+/+}$ and *Mb1-Cre*$^{+}$ *Cxcr4*$^{fl/-}$ mice were infected with influenza and their medLNs were harvested on day 11 p.i.. (A) GC polarization and centrocyte positioning were determined by IHC. (B) Representative FACS plot indicating the frequencies of CD83^{lo}CD86^{lo} centroblasts and CD83^{hi}CD86^{hi} centrocytes in CXCR4-deficient and control mice. (C) Summary of data with each dot representing a single mouse.

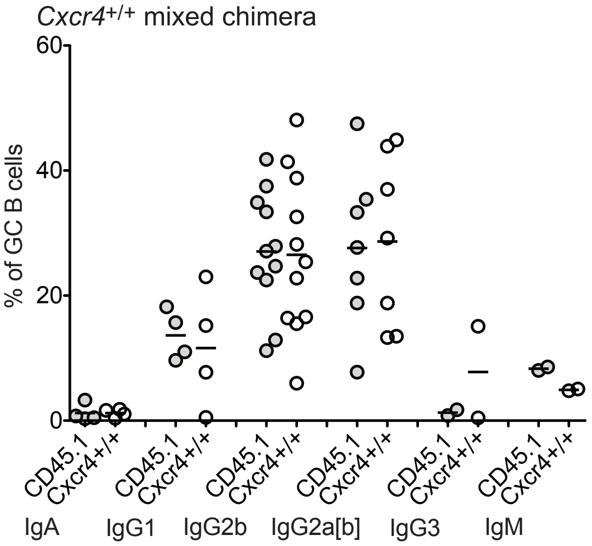
Cxcr4+/+ mixed chimera
% of GC B cells
60
40
20
0
CD45.1
Cxcr4+/+
IgA
IgG1
IgG2b
IgG2a[b]
IgG3
IgM

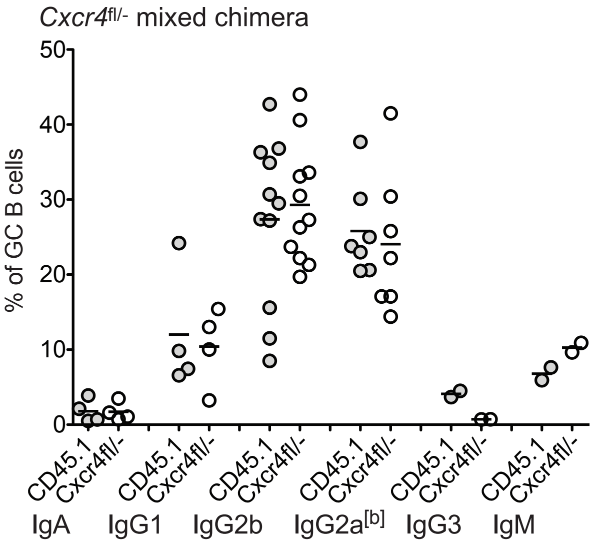
Cxcr4fl/- mixed chimera
% of GC B cells
50
40
30
20
10
0
CD45.1
Cxcr4fl/-
IgA
IgG1
IgG2b
IgG2a[b]
IgG3
IgM

Figure S3. Normal antibody class switching by CXCR4-deficient GC B cells, Related to Figure 5.

Surface expression of specific antibody isotypes on WT CD45.2$^+$ and CXCR4-deficient or control GC B cells from mixed chimeras was determined by FACS. Data are pooled from multiple experiments with each dot representing a single mouse.

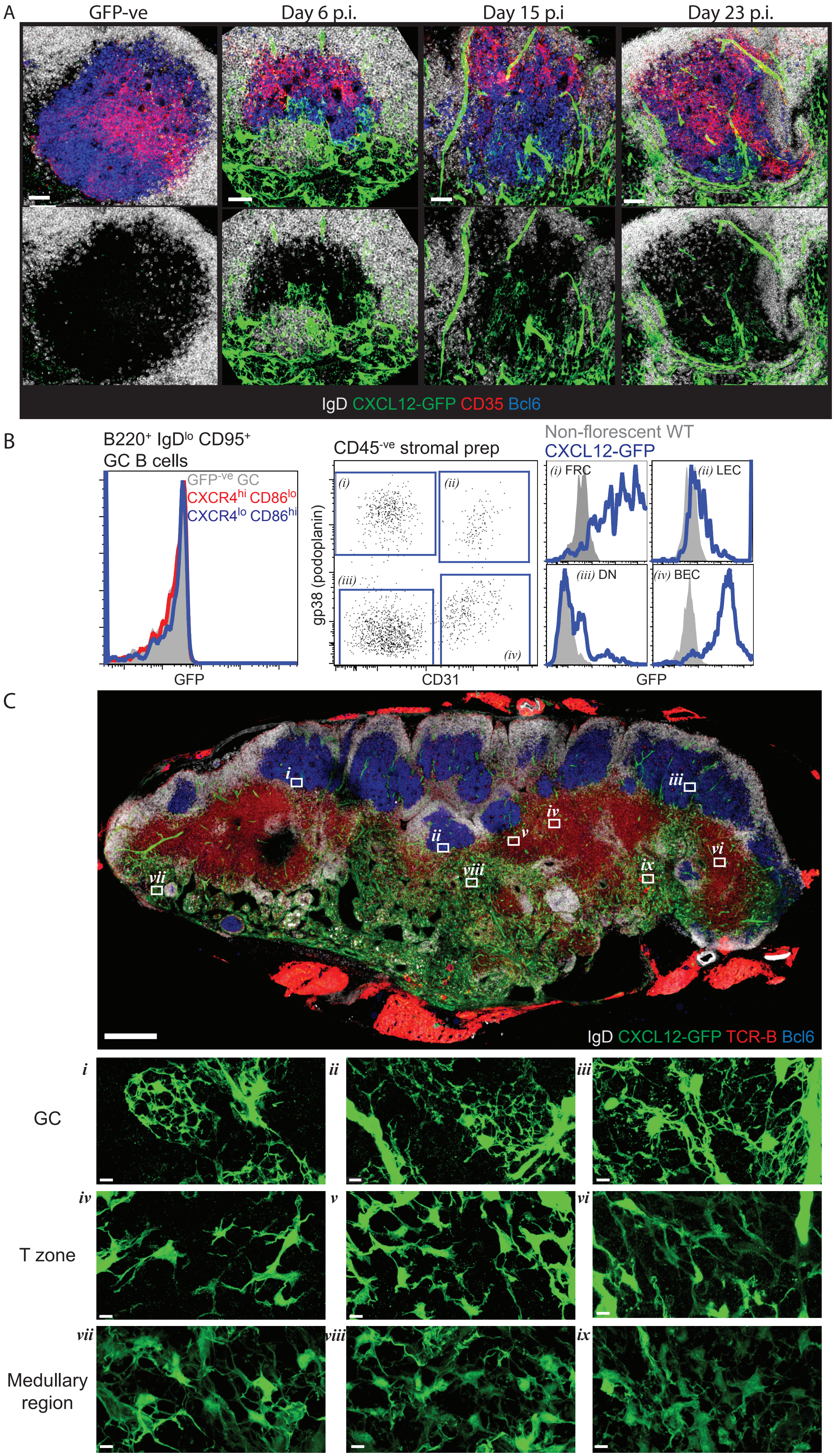
A
GFP-ve
Day 6 p.i.
Day 15 p.i
Day 23 p.i.
IgD CXCL12-GFP CD35 Bcl6
B
B220+ IgDlo CD95+
GC B cells
GFP-ve GC
CXCR4hi CD86lo
CXCR4lo CD86hi
GFP
CD45-ve stromal prep
gp38 (podoplanin)
CD31
(i)
(ii)
(iii)
(iv)
Non-florescent WT
CXCL12-GFP
(i) FRC
(ii) LEC
(iii) DN
(iv) BEC
GFP
C
i
ii
iii
iv
v
vi
vii
viii
ix
IgD CXCL12-GFP TCR-B Bcl6
GC
T zone
Medullary
region

Figure S4. Characterization of DZ CXCL12-expressing reticular cells, related to Figure 7.

(A*) Cxcl12-gfp* heterozygous mice were infected with influenza virus and their medLN GCs examined by confocal microscopy for the presence of CXCL12-expressing reticular cells at various time points p.i.. MedLNs from WT GFP^{-ve} mice were stained with the same antibodies to confirm the specificity of the anti-GFP reagent. (B) GC B cells (left) and CD45 negative stromal cells (right) were assessed for GFP expression at ~2 weeks p.i.. (C) A cross-section of a medLN from day 15 p.i., indicating the location of the higher magnification images shown below. Images highlight differences in CXCL12-expressing cell morphology in the GC DZ, the T zone and the medullary regions.

Example 1

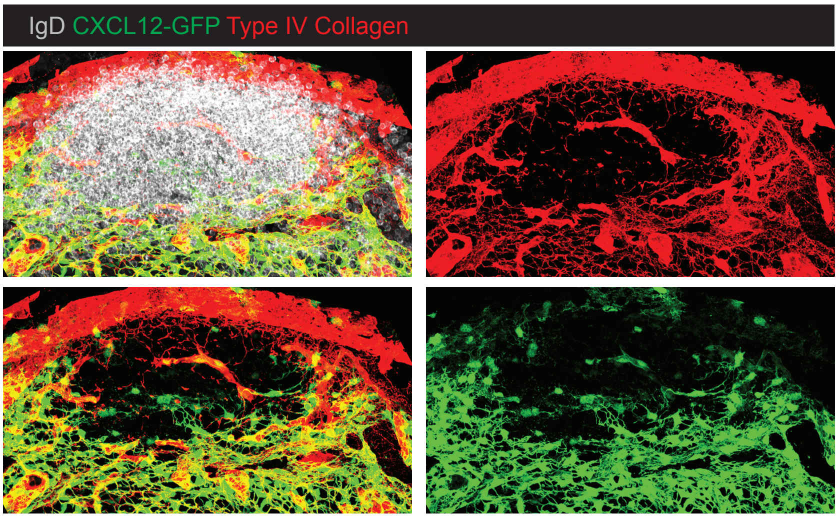

Example 2

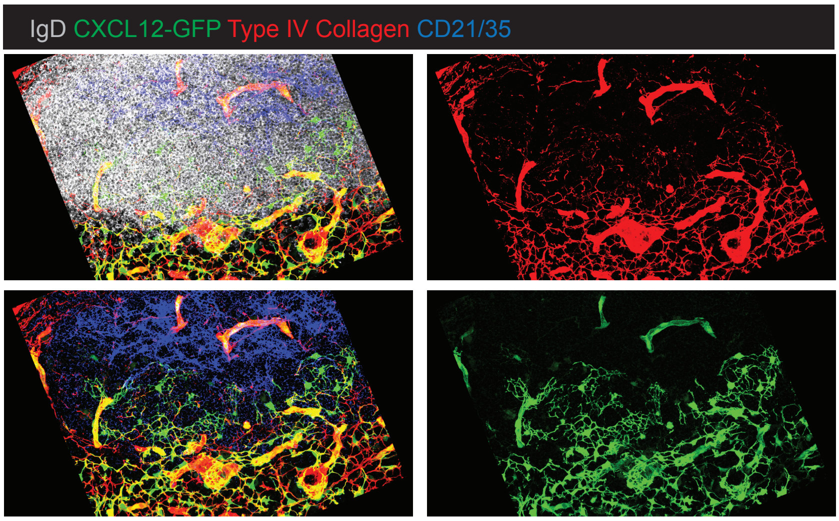

Example 3

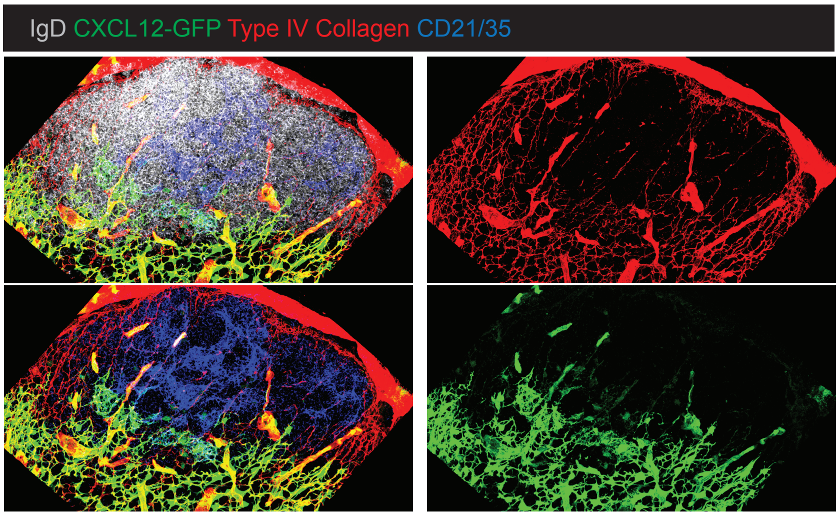

Figure S5. Additional examples of type IV collagen and CXCL12-GFP staining in pLN primary follicles, related to Figure 7.

Scale bars are 40uM (A), 30uM (C, full LN) and 7uM (C, small images).
