# Supplementary figures and images for: Germinal Center Centroblasts Transition to a Centrocyte Phenotype According to a Timed Program and Depend on the Dark Zone for Effective Selection
Source: Immunity. 2013 Nov 14;39(5):912–24. doi: 10.1016/j.immuni.2013.08.038 (PMC3828484; doi:10.1016/j.immuni.2013.08.038)

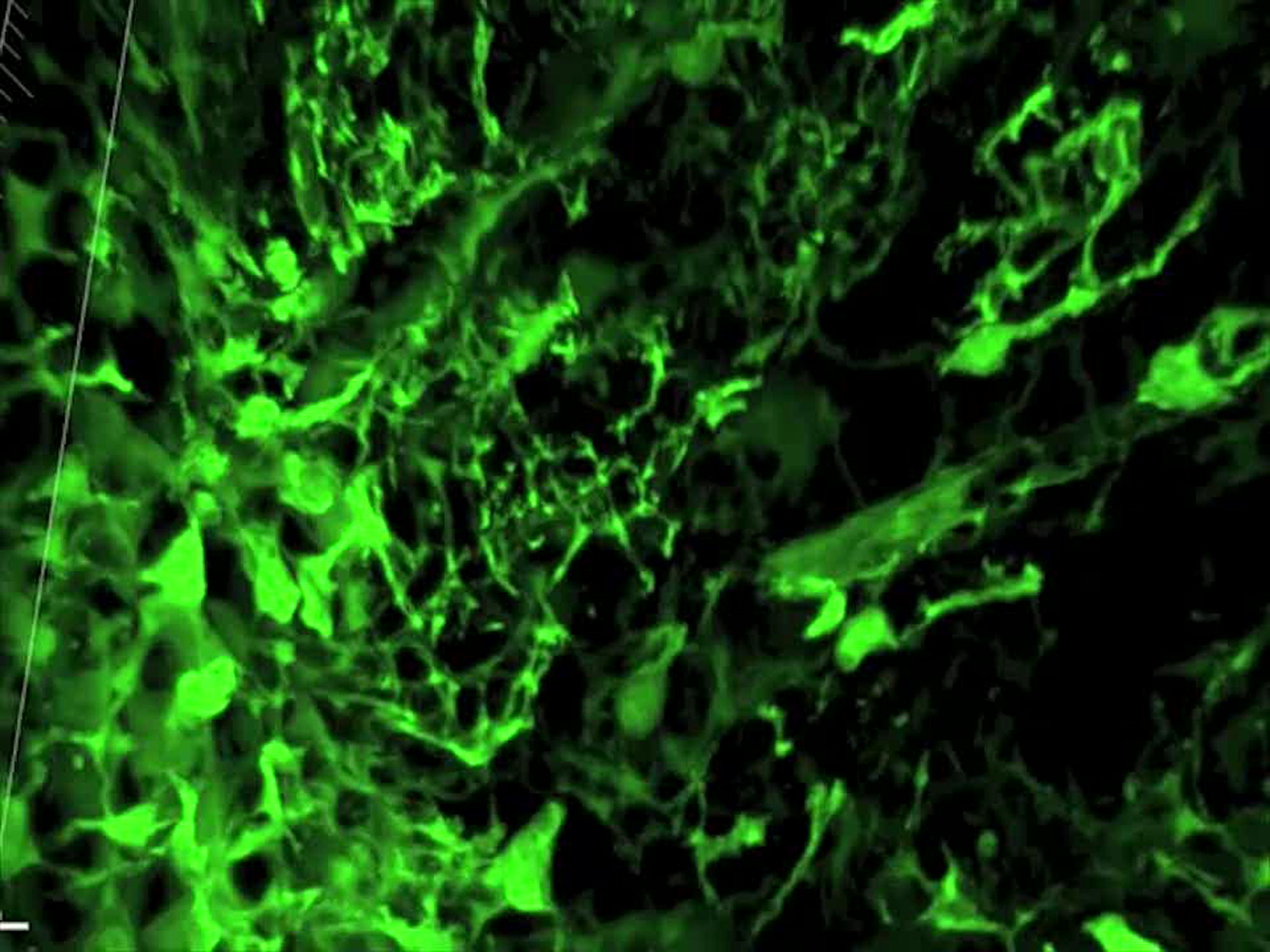

Supplement: Movie S1. DZ CXCL12-Expressing Reticular Cells — PP and influenza induced GCs in Cxcl12-gfp mice were stained with fluorescent antibodies and examined by confocal microscopy. Movie shows a selection of representative examples. The antibodies used are indicated for each example. Related to Figure 7. [file mmc2.jpg]
